# Expert opinion on treatment patterns in metastatic colorectal cancer management in Eastern Europe

**DOI:** 10.1186/s12919-026-00369-1

**Published:** 2026-03-23

**Authors:** László Torday, Alinta Hegmane, Biljana Kukić, Edita Baltruškevičienė, Elina Sivina, Ioana Luca, Juraj Prejac, Marija Ristić, Martina Reberšek, Nikola Milašević, Rossitza Krasteva Ruseva, Saša Jungić, Velko Todorov Minchev, Tina Roblek, Viktor Uršič

**Affiliations:** 1 Department of Oncotherapy, University of Szeged, Szeged, Hungary; 2 County Oncology Center, Békés County Central Hospital, Gyula, Hungary; 3 Riga East University Hospital, Oncology Center of Latvia, Hipokrāta Iela 4, Rīga, Latvia; 4 University of Latvia, Rīga, 1586 Latvia; 5 Department of Medical Oncology, Oncology Institute of Vojvodina, Put Doktora Goldmana 4, Sremska Kamenica, 21204 Serbia; 6 Faculty of Medicine Novi Sad, University of Novi Sad, Hajduk Veljkova 3, Novi Sad, 21000 Serbia; 7 National Cancer Institute, Santariškių G. 1, Vilnius, Lithuania; 8 Fundeni Clinical Institute, Fundeni Street 258, Bucharest, Romania; 9 Department of Oncology, University Hospital Centre Zagreb, Ulica Mije Kišpatića 12, Zagreb, Croatia; 10 School of Dental Medicine, University of Zagreb, Gundulićeva Ulica 5, Zagreb, Croatia; 11 Institute of Oncology and Radiology of Serbia, Pasterova 14, Belgrade, Serbia; 12 Department of Medical Oncology, Institute of Oncology Ljubljana, Zaloška Cesta 2, Ljubljana, Slovenia; 13 Medical Faculty, University of Ljubljana, Vrazov Trg 2, Ljubljana, 1000 Slovenia; 14 Clinic for Medical Oncology, Institute for Oncology, Clinical Centre of Montenegro, Podgorica, Montenegro; 15 University Hospital Sofiamed, 16 G.M. Dimitrov Blvd., Sofia, 1113 Bulgaria; 16 Multiprofile Hospital for Active Treatment University Hospital, 100 Georgi Benkovski Str., Panagyurishte, 4500 Bulgaria; 17 Oncology Clinic, University Clinical Center, Republic of Srpska, Str 12 Beba Bb, 78 000 Banjaluka, Bosnia and Herzegovina; 18 Faculty of Medicine, University in Banjaluka, Str Save Mrkalja 14, Banjaluka, 78 000 Bosnia and Herzegovina; 19 SofiaMed University Hospital, Sofia, 1113 Bulgaria; 20 Takeda Pharmaceuticals d.O.O., Bleiweisova Cesta 30, Ljubljana, Slovenia

**Keywords:** Eastern Europe, Colorectal cancer, Metastatic, Treatment patterns

## Abstract

Colorectal cancer (CRC) is the third most prevalent malignancy globally and the second leading cause of cancer-related mortality. Approximately 15%–30% of patients are diagnosed with metastatic CRC (mCRC), and despite increasing survival rates, mCRC remains fatal. There are notable variations in the incidence and mortality rates of CRC across European countries, which may be attributed to the differences in lifestyle patterns and variations in cancer diagnosis and management practices. Standard initial treatments for mCRC include chemotherapeutic agents and targeted therapies. This report presents the findings of a virtual meeting held from 21 January to 2 February 2025. The meeting included healthcare professionals from 10 Eastern European countries (Bosnia and Herzegovina, Bulgaria, Croatia, Hungary, Latvia, Lithuania, Montenegro, Romania, Serbia, and Slovenia). The experts shared their opinions on various topics such as the current clinical practice and existing clinical challenges in the treatment of patients with mCRC, factors influencing treatment choices, and the place of fruquintinib therapy in the management of mCRC. The discussion was facilitated using open-ended questions. The primary objective of this article was to provide an overview of the experts’ opinions and experiences related to the current treatment approaches to mCRC management, with a focus on fruquintinib in Eastern Europe. The experts identified the need for molecular and genetic profiling, multidisciplinary coordination, limited availability of and access to targeted and newly approved medications, and lack of reimbursement as key challenges in managing patients with mCRC in these regions. The experts also highlighted the importance of patient education and participation in regional and international clinical trials of new therapeutics. They also noted the need for generating real-world evidence on the safety and efficacy of fruquintinib, its comparative efficacy versus other later-line therapies, its efficacy as an earlier line of therapy, and its sequencing in the current treatment practices. Furthermore, despite the lack of direct experience with fruquintinib, most experts acknowledged its potential efficacy as a later-line therapy for mCRC and considered its effectiveness to be comparable to that of other third-line treatments.

## Introduction

Colorectal cancer (CRC) is the third most prevalent malignancy globally, with an annual incidence of over 1.9 million cases, and accounts for approximately 10% of all cancer cases [1]. It is the second leading cause of cancer-related mortality [1]. According to the European Cancer Information System, CRC accounted for 13% of all new cancer cases and 12% of all cancer-related deaths in Europe in 2022 [2]. Furthermore, there are varying trends in the incidence and mortality of CRC across European countries, with reductions in both the incidence and mortality rates in countries with well-established screening programmes [3]. Furthermore, European countries vary in screening programme implementation, including scope, participation rates, and techniques, with the lowest participation rates observed in countries with the lowest levels of education and income [4]. The variations in cancer mortality rates across Western and Eastern Europe could be due to differences in lifestyle patterns and variations in cancer diagnosis and management practices in these two regions [5]. Moreover, findings from the EUROCARE studies revealed significant regional variations in age-adjusted 5-year CRC survival rates, with Northern and Central European countries showing the highest survival rates but Eastern European countries exhibiting the lowest [6]. Despite the implementation of screening programmes, approximately 15%–30% of patients with CRC are diagnosed with metastatic disease, and up to 50% of these patients with initially localised disease develop metastases [7]. The liver, lungs, peritoneum, and distant lymph nodes are the most frequent sites of metastases [7]. Despite increasing survival rates, metastatic CRC (mCRC) remains a lethal disease with a 5-year survival rate of approximately 15% [8].

The European Society for Medical Oncology (ESMO) guidelines outline advancements in the areas of diagnosis, staging, and therapeutic interventions for patients with mCRC, which have significantly influenced the current ‘state-of-the-art’ treatment methodologies and offer recommendations for the holistic management of individuals diagnosed with mCRC [7]. The standard initial treatments for mCRC include chemotherapeutic agents and targeted therapies, as appropriate, and third- or later-line treatment options include trifluridine/tipiracil and regorafenib (a multikinase inhibitor) [7]. The treatment approach for patients with mCRC follows a continuum of care, incorporating an integrated sequential comprehensive treatment strategy to achieve optimal outcomes [9].

The aim of this report was to provide an overview of experts’ opinions and experiences in the current treatment approaches to mCRC management, with a focus on fruquintinib in Eastern Europe. Insights from participating healthcare professionals from 10 Eastern European countries (Bosnia and Herzegovina, Bulgaria, Croatia, Hungary, Latvia, Lithuania, Montenegro, Romania, Serbia, and Slovenia) were collected during a virtual meeting held from 21 January to 2 February 2025. Topics such as current clinical practices and their associated challenges, factors influencing treatment choices, and the place of fruquintinib therapy in the management of mCRC were discussed. The discussion was facilitated using open-ended questions.

## Treatment approaches

According to the experts who participated in the meeting, the current clinical practice for mCRC management adheres to the ESMO guidelines and includes a combination of surgery, chemotherapy, and targeted therapies. Strategies encompass the integration of surgical interventions, chemotherapy regimens (such as FOLFOX [folinic acid, fluorouracil, and oxaliplatin] and FOLFIRI [folinic acid, fluorouracil, and irinotecan]), and targeted therapeutic agents (bevacizumab, cetuximab, and panitumumab). For cases characterised by microsatellite instability (MSI)-high/deficient mismatch repair (MSI-H/dMMR), immunotherapeutic approaches (pembrolizumab, nivolumab, or nivolumab/ipilimumab combination) are also considered. Table 1 summarises country-specific findings regarding mCRC management.

**Table 1:** Summary of country-specific information on mCRC management

Country	Molecular testing and TAT	Reimbursement and access	Later-line therapies in practice	MDT and care organisation
Bosnia and Herzegovina	• No in-house MSI • RAS/BRAF by PCR • Liquid biopsy not available/reimbursed • NGS not available locally (can be performed abroad privately)	• Limited access to and availability of newer agents • Immunotherapy not reimbursed • Emphasis on the need for NGS and early-access/clinical trials	• Trifluridine/tipiracil (± bevacizumab) is the main option • Regorafenib and fruquintinib not available • 3L or 4L: selective anti-EGFR rechallenge for RAS-WT or rechallenge with chemotherapy	• MDTs function in university/clinical centres; decision-making via MDT is emphasised
Bulgaria	• KRAS/NRAS and BRAF testing supported (often via pharma) • MSI and NGS typically paid by patients • Biopsy of new lesions and repeat molecular work-up encouraged	• 1L/2L regimens with bevacizumab/anti-EGFR reimbursed by the NHIF • Off-label not reimbursed • Fruquintinib not available • Inclination towards an early-access programme	• Regorafenib or trifluridine/tipiracil (± bevacizumab) in 3L + • Clinicians report no access to fruquintinib and anticipate benefit once available	• MDT approach with tumour board discussions reported
Croatia	• MMR testing mandatory before 1L • NGS available to all patients with metastases via a special fund • DPYD and UGT1A often performed in expert’s clinical centre • Liquid biopsy used to triage EGFR rechallenge	• Comprehensive genomic profiling and approved therapies funded via a dedicated fund • Fruquintinib via an early-access programme only	• Trifluridine/tipiracil ± bevacizumab preferred in 3L • Regorafenib used when mechanism switch needed • Fruquintinib considered after prior options (EAP currently)	• MDT opinion mandatory • Caution about low-volume centres proceeding without full MDT/molecular work-up
Hungary	• For mCRC: MMR IHC (10–15 WD) • RAS/BRAF by NGS (within 3 weeks) • MSI by PCR may take 2 months; all molecular tests reimbursed • Liquid biopsy available in ≥ 2 centres for EGFR rechallenge	• Immunotherapy reimbursed after a case-by-case review • Calls for expanded access programmes and faster individual financing (incl. for fruquintinib) • Access variability and administrative burden noted	• Shift towards trifluridine/tipiracil + bevacizumab; regorafenib use curtailed due to toxicity/logistics • Fruquintinib positioned post-trifluridine/tipiracil or regorafenib (per FRESCO-2)	• Upon progression on 1st‑line therapy, MDT consultation is mandatory before initiating 2nd‑line treatment
Latvia	• RAS/BRAF by PCR tested in one laboratory (TAT up to 10 WD) • NGS occasional (TAT 2 months) • Liquid biopsy not reimbursed	• Immunotherapy not reimbursed until March 2025 • Anti-EGFR reimbursed only in 1L; BRAF/HER2 agents not reimbursed • Fruquintinib not available	• 3L: Trifluridine/tipiracil + bevacizumab preferred • Regorafenib reimbursed (can be used in 3L) • Anti-EGFR rechallenge not reimbursed • Fruquintinib anticipated post-trifluridine/tipiracil once available	• Centralised care in 4 centres; MDT mandatory pre-surgery and for targeted drug starts/line changes
Lithuania	• MMR routine (hospital-funded) • RAS PCR (pharma-supported) • BRAF PCR not covered • Small-panel NGS planned for reimbursement	• Immunotherapy not reimbursed for MSI-H • No anti-BRAF/HER2, no fruquintinib • Bevacizumab with trifluridine/tipiracil often patient-paid	• Trifluridine/tipiracil and regorafenib reimbursed (trifluridine/tipiracil often the first 3L choice and regorafenib later due to its toxicity) • No fruquintinib access	• MDT mandatory • Treating physician decides unless a complex case • Treatment is centralized to five institutions
Montenegro	• No reflex MSI; RAS/BRAF by PCR (TAT 2 weeks, reimbursed) • NGS used for wider panels • Liquid biopsy not available/reimbursed • DPYD not routine	• Later-line access limited • Calls for better diagnostics (incl. liquid biopsy) and more drugs in later lines of therapy	• Trifluridine/tipiracil + bevacizumab available • Fruquintinib and regorafenib not available • Fruquintinib considered valuable if accessible	• MDT crucial • Systemic therapy/radiation centralised at the Institute of Oncology
Romania	• IHC/PCR reimbursed for advanced disease • NGS not routinely settled • Liquid biopsy for RAS sometimes supported post-progression by the industry	• Immunotherapy for MSI-H reported as becoming reimbursed • Standard agents generally accessible • Fruquintinib not available (patients may buy it abroad)	• Trifluridine/tipiracil + bevacizumab newly adopted • Regorafenib less favoured due to tolerability issues • Fruquintinib not yet accessible	• Long-standing MDT process with periodic re-evaluation
Serbia	• MMR/MSI and NGS not routine/reimbursed; RAS/BRAF by PCR (TAT within 5–7 days) • KRAS/NRAS testing mandatory • BRAF and MSI tests available but not reimbursed • Liquid biopsy not reimbursed (limited to EGFR rechallenge projects)	• Immunotherapy not reimbursed • BRAF/HER2 therapies not reimbursed • Regorafenib and fruquintinib usable only under special circumstances	• 3L: trifluridine/tipiracil (typically without bevacizumab) • 4L: regorafenib and/or fruquintinib in select cases • Limited experience with fruquintinib	• Centralised care; MDT mandatory before treatment decisions
Slovenia	• All testing reimbursed; NGS hotspot TAT 1 week (others ≥ 3 weeks) • Liquid biopsy desirable for rechallenge	• All testing and drugs reimbursed by the National Health Insurance • Fruquintinib is available only via early access • Waiting for fruquintinib reimbursement; otherwise, broad access to testing/drugs	• Trifluridine/tipiracil + bevacizumab (3L), regorafenib (4L) • Fruquintinib via EAP currently	• All patients on MDT board before their first treatment; decisions consider ECOG, comorbidities, prior lines, and availability

*1L* first line of therapy, *2L* second line of therapy, *3L* third line of therapy, *4L* fourth line of therapy, *BRAF* v-Raf murine sarcoma viral oncogene homologue B, *DPYD* dihydropyrimidine dehydrogenase, *EAP* early access programme, *ECOG* Eastern Co-operative Oncology Group, *EGFR* epidermal growth factor receptor, *HER2* human epidermal growth factor receptor 2, *IHC* immunohistochemistry, *KRAS* Kirsten rat sarcoma viral oncogene homologue, *mCRC* metastatic colorectal cancer, *MDT* multidisciplinary team, *MMR* mismatch repair, *MSI* microsatellite instability, *MSI-H* microsatellite instability-high, *NGS* next-generation sequencing, *NHIF* National Health Insurance Fund, *NRAS* neuroblastoma rat sarcoma viral oncogene homologue, *PCR* polymerase chain reaction, *RAS* rat sarcoma, *RAS-WT* rat sarcoma wild-type, *TAT* turnaround time, *UGT1A* uridine diphosphate glucuronosyltransferase family 1 member A, *WD* work day

## Factors influencing treatment selection

### Patient characteristics

The management of mCRC is personalised according to patient-specific variables, including tumour genetics (Kirsten rat sarcoma [RAS] viral oncogene homologue [KRAS], neuroblastoma RAS viral oncogene homologue [NRAS], v-Raf murine sarcoma viral oncogene homologue B [BRAF], and MSI/mismatch repair [MSI/MMR] status), tumour characteristics (location and resectability of metastases), overall patient health, existing comorbidities, and personal preferences.

All experts agreed that patient profiles play a crucial role in treatment decisions for mCRC, as each patient’s unique characteristics, including their clinical, genetic, and psychosocial factors, significantly influence the choice of therapy and the overall management strategy. The key elements of patient profiling that are considered in treatment decisions for mCRC are molecular profiling (RAS, especially KRAS and NRAS status; BRAF; and MMR), tumour sidedness, Eastern Co-operative Oncology Group performance status, age, disease extent, access to targeted drugs, and response and tolerability to prior treatments.

A multidisciplinary team (MDT) and/or the treating physician generally decide a patient’s eligibility for intensive treatment. Collaborative discussions within the MDT and the identification of novel biomarkers, such as neurotrophic tyrosine receptor kinase, human epidermal growth factor receptor 2 (HER2), and KRAS G12C, significantly influence therapeutic strategies. Additionally, factors such as potential adverse events, a patient’s ability to tolerate various treatments, and access to advanced diagnostics and innovative therapies are integral to the decision-making process. Educating patients about the available treatment modalities and their associated side effects is essential for fostering informed choices, which necessitates a careful balance between evidence-based guidelines and individual patient preferences.

### Molecular testing

Although molecular testing capabilities are improving with the gradual rollout of next-generation sequencing (NGS) panels, the experts highlighted persistent challenges such as long turnaround times, inconsistent reimbursement, and limited availability of liquid biopsy. Access to individual biomarker tests has been shown to be impeded in Eastern European countries owing to the limited public reimbursement for diagnostic testing and variability in order rates [10]. Moreover, less than 75% of laboratories in Eastern European countries have access to NGS technologies, either internally or through referral [10]. These delays may postpone treatment initiation and complicate timely decision-making for targeted therapies. Standardising reflex testing and ensuring reimbursement for comprehensive molecular profiling have been identified as urgent priorities for optimising care pathways.

## Late-line treatment in mCRC

The experts opined that, in the later stages of treatment for mCRC, the emphasis shifts towards the management of the disease, alleviation of symptoms, improvement of survival outcomes, and maintenance of quality of life. Treatment decisions are guided by considerations such as the patient’s prior responses to therapy, adverse effects experienced, performance status, existing comorbidities, availability of medications, and current guideline recommendations.

However, patients’ preferences are of paramount importance in determining management approaches. There are several approaches such as re-administration of chemotherapy, molecular profiling-led targeted therapies, and participation in clinical trials. The clinical practice related to treatment sequencing in most countries adheres to the recommendations of the ESMO guidelines, but these should be individualised based on previous treatment responses, the patient’s conditions, and drug availability.

For patients with mCRC, drug rechallenging necessitates the evaluation of previous chemotherapy efficacy, progression-free survival (PFS), the patient’s functional status, and molecular genetics such as RAS/BRAF profiles. The combination of trifluridine/tipiracil with bevacizumab is preferred over regorafenib because of its enhanced tolerability and superior PFS, although regorafenib remains a viable alternative when a change in the mechanism of action is deemed necessary. In Lithuania, while bevacizumab combination therapy is not reimbursed, trifluridine/tipiracil monotherapy is also administered. Rechallenge may be justified by the success of anti-epidermal growth factor receptor (EGFR) therapeutics, especially following a substantial treatment-free interval, facilitating the possibility of sensitivity reinstatement. Maintenance methodologies, reinstatement of pharmacological agents after maintenance therapy, and rechallenge determinations differ by region and available evidence, accentuating the significance of individualised patient evaluation and the prospective utility of liquid biopsies for informed clinical decision-making.

CRC treatments are conducted in centralised cancer centres in Hungary, Latvia, Serbia, and Lithuania. In Croatia, treatments excluding radiotherapy are conducted in regional centres, whereas in Bosnia and Herzegovina, specific oncological treatments are conducted exclusively in university hospitals and clinical centres where oncology MDTs are onboard. In Montenegro, only the Institute of Oncology provides systemic chemotherapy and radiation therapy for patients with mCRC. Decision-making through MDT is mandatory in all countries in Eastern Europe. The decision-making process is also shaped by molecular diagnostic findings and policies governing drugs and testing reimbursement at the national level.

## Unmet needs in treatment options for managing patients with mCRC

**Fig. 1:**
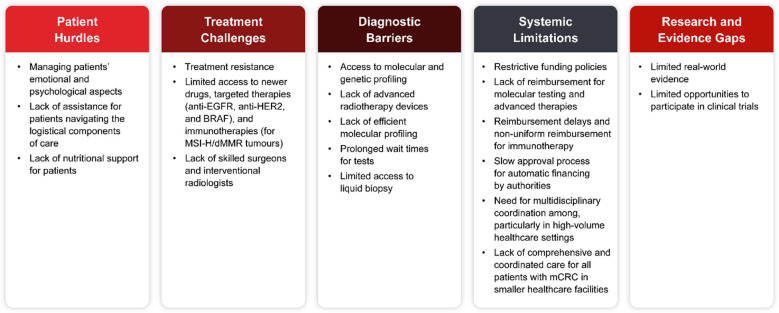
Challenges associated with the management of patients with mCRC in Eastern Europe. BRAF, v-Raf murine sarcoma viral oncogene homologue B; dMMR, deficient mismatch repair; EGFR, epidermal growth factor receptor; HER2, human epidermal growth factor receptor 2; mCRC, metastatic colorectal cancer; MSI-H, microsatellite instability-high

Management of patients with mCRC presents several challenges, such as patient hurdles, treatment resistance, molecular and genetic profiling, multidisciplinary coordination, limited availability of and access to targeted treatment, and monetary issues due to reimbursement challenges (Fig. 1).

In Lithuania, there is limited access to newer drugs and targeted therapies (anti-HER2 and anti-BRAF, and immunotherapy). Similarly, there is limited access to anti-EGFR, anti-HER2, and anti-BRAF therapies in Latvia, with anti-EGFR agents being the only reimbursed targeted therapy when administered as a first-line treatment. Immunotherapies (for MSI-H/dMMR tumours) were not reimbursed in Latvia until March 2025. Nevertheless, there is limited access to such immunotherapies, with pembrolizumab being the only one reimbursed as a first-line treatment for MSI-H/dMMR mCRC. In Hungary, immunotherapy is reimbursed only following a centralised, case-by-case review. Although pembrolizumab is accessible via this strategy, the availability of ipilimumab/nivolumab remains uncertain. Reimbursement for immunotherapy is not uniform across all countries, with no support in Bosnia and Herzegovina, Lithuania, and Serbia. For instance, despite being considered as a potentially effective treatment option, the availability of fruquintinib remains limited. Challenges exist in ensuring comprehensive and coordinated care for all patients with mCRC, particularly in small healthcare facilities. The lack of sufficient modern radiotherapy devices in public hospitals and advanced radiotherapy (stereotactic body radiation therapy and brachytherapy) is one of the main unmet needs in mCRC treatment in Croatia. Additionally, the paucity of skilled surgeons and interventional radiologists and the slow approval process for automatic financing by authorities are unmet needs in Hungary.

The limited opportunities to participate in clinical trials are another unmet need in Latvia, Lithuania, and Slovenia. Some experts (Bulgaria, Serbia, and Slovenia) stressed that diagnostic challenges, particularly the lack of efficient molecular profiling, long wait times, and limited access to liquid biopsy due to reimbursement issues, pose challenges to appropriate and timely treatment.

Molecular marker testing is reimbursed in Croatia, Hungary, Latvia, and Slovenia. MSI/MMR testing is not routinely conducted in Latvia, Serbia, Montenegro, and Bosnia and Herzegovina, but it is recommended for all patients in Romania and now routinely conducted in Hungary. In Lithuania, NGS is reimbursed, and MMR testing is recommended and performed upon request. In Slovenia, younger patients are frequently diagnosed with advanced disease owing to their exclusion from screening protocols, resulting in delayed diagnosis.

Managing the emotional and psychological aspects associated with the treatment of patients with mCRC constitutes a persistent challenge in most participating countries. The provision of sufficient emotional support and the inclusion of caregivers within the treatment framework are fundamental components of holistic care. Furthermore, assisting patients in navigating the logistical components of care (such as transportation, medical consultations, computed tomography [CT] scans, and blood examinations) introduces an additional challenge. The experts highlighted the role of national registries as a platform for recording the quality of treatment outcomes and generating real-world data from a large population of patients treated for mCRC, identifying eligible patients for clinical trials, and devising new treatment protocols. For this purpose, a national oncology database has been introduced in Croatia. Similarly, a central registry, mainly providing epidemiological data, also exists in Hungary.

## Approaches to improve the management of mCRC

The experts discussed the need for improved patient education, quicker access to molecular genetic tests, implementation of liquid biopsy, reimbursement for various cancer treatments, expanded access programmes for new medications, enhanced data collection, and importance of multidisciplinary approaches in managing mCRC. The experts emphasised advocating for better drug availability, increased influence of oncologists in healthcare decisions, and more efficient administrative processes. Collaborative decision-making is essential, with discussions within an MDT playing a crucial role in guiding therapeutic strategies across various stages that consider the condition of the patients, genomic characteristics of the tumours, and availability of effective drugs, along with the corresponding reimbursement frameworks in the region.

## Evaluating the effectiveness of various treatment options for mCRC

Evaluating treatment responses involves clinical examinations, laboratory tests (including tumour markers such as carcinoembryonic antigen [CEA] and carbohydrate antigen 19–9 [CA19-9]), and imaging (CT, magnetic resonance imaging, and positron emission tomography) every 3–6 months, adapted to the patients’ progression. Decisions are based on clinical trial results, real-world evidence, response rates, impact on overall survival, and patients’ discussions regarding expectations. Real-world data collection, despite challenges, enhances treatment choices and is complemented by patient-reported outcomes for quality of life, although this is not always a standard practice.

## Fruquintinib as a potential treatment in mCRC

Fruquintinib is a novel, highly selective, well-tolerated, and potent oral tyrosine kinase inhibitor of vascular endothelial growth factor receptors (VEGFRs) 1, 2, and 3 [11, 12]. The United States Food and Drug Administration recently approved its use in patients with mCRC experiencing disease progression during or after chemotherapy, anti-VEGF biological therapy, anti-EGFR biological therapy (if RAS wild-type), and treatment with either trifluridine/tipiracil or regorafenib [13]. Fruquintinib inhibits tumour proliferation by obstructing angiogenesis and curtailing its spread through the reduction of lymphangiogenesis [12, 14]. It also exhibits weak inhibition of receptor tyrosine kinases, such as rearranged during transfection (RET), fibroblast growth factor receptor 1 (FGFR-1), and c-KIT kinases, which are major mediators of tumour cell proliferation [15].

**Fig. 2:**
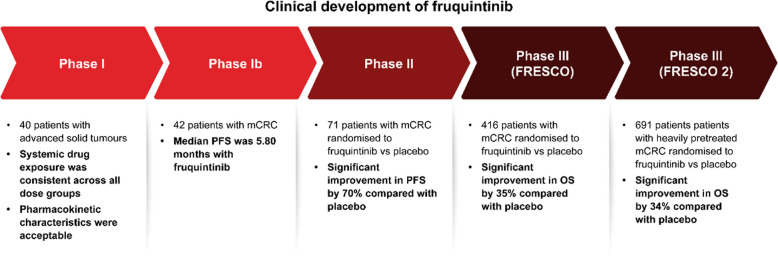
Clinical developmental history of fruquintinib in mCRC [16, 17, 24, 25]. mCRC, metastatic colorectal cancer; OS, overall survival; PFS, progression-free survival

Fruquintinib and new combinations of treatments offer potential options for patients with mCRC who are molecularly selected and for all-comer patients with extensive prior treatments [12]. The clinical development of fruquintinib is summarised in Fig. 2. The FRESCO (NCT02314819) [16] and FRESCO 2 (NCT04322539) [17] clinical trials provided clinical evidence for the therapeutic relevance of fruquintinib in patients with chemorefractory mCRC [18, 19]. The safety profile of fruquintinib is comparable to that of other VEGFR inhibitors; however, its high specificity for VEGFR-1, 2, and 3 may result in some differences compared with other more broadly acting inhibitors [20]. Furthermore, for oral administration of chemotherapy, there were noted improvements in both treatment satisfaction and quality of life, with higher treatment satisfaction being associated with greater adherence [21]. Real-world data have shown promising outcomes [22, 23].

Regorafenib and fruquintinib are oral VEGFR inhibitors used in later-line mCRC therapy; however, fruquintinib shows a promising balance between efficacy and safety for patients with heavily pre-treated mCRC, with similar or better PFS and overall survival than with regorafenib [26–28]. Moreover, the experts noted that fruquintinib may offer a more favourable tolerability profile than regorafenib, especially the more manageable hypertension versus dermatologic toxicity often associated with regorafenib. Therefore, selecting between these agents remains a highly relevant consideration in clinical practice across the region.

## Expert opinion on the place of fruquintinib therapy in the management of mCRC

Most experts lacked direct experience with fruquintinib; however, they recognised its potential efficacy, as indicated by the results of the FRESCO trials. All experts discussed its effectiveness and tolerance as a late-line mCRC treatment. Based on the available data on fruquintinib, the experts suggested that it has better efficacy in terms of PFS than regorafenib and is comparable to other treatment options. They underscored that an acceptable toxicity profile and prior treatment failure were key factors for choosing fruquintinib over other late-line mCRC treatments. Other factors included performance status, enhanced survival, benefits of oral administration, and improved quality of life. The experts emphasised the importance of considering patient conditions, preferences, and prior treatment effects. Patients diagnosed with a slowly progressive disease, unresponsive to multiple standard treatments, and without significant cardiovascular complications or other critical risk factors for adverse events are ideal candidates for fruquintinib treatment.

The presence of pre-existing medical conditions and comorbidities is a crucial consideration when evaluating the administration of fruquintinib. Precautionary measures must be implemented for patients with hypertension and renal, hepatic, and/or thyroid dysfunction, with consideration of dose modulation or treatment cessation based on the patient’s clinical response.

The limited availability of advanced anti-cancer drugs further restricts equitable access to healthcare in Eastern Europe [29]. Across all participating countries, lack of reimbursement, reimbursement delays, and restrictive funding policies were consistently cited as the primary obstacles that limited patient access to fruquintinib treatment. Significant heterogeneity exists in reimbursement policies across Eastern Europe, which delays or prevents access to fruquintinib for many eligible patients. Some countries have introduced partial coverage for molecular testing or later-line agents, whereas others rely heavily on out-of-pocket payments or pharma-sponsored programmes.

The experts across the region consistently highlighted that reimbursement delays and restricted access are the primary obstacles to the adoption of new treatments such as fruquintinib. Although the main chemotherapy medications for the management of CRC are widely available and generally subsidised in Eastern Europe, more expensive advanced therapies, such as regorafenib, are rarely accessible other than at full cost in Eastern Europe [30], with the exception being Lithuania, where regorafenib is reimbursed. In some regions, fruquintinib is available only through early access programmes or private purchases, thus creating disparities in care. These challenges further widen the gap between guideline recommendations and their practical implementation, particularly in resource-constrained settings. Despite the approval of many novel drugs, such as fruquintinib, national-level negotiations and administrative processes often result in prolonged timelines before patients can access treatment. The time span between drug registrations and national decisions on reimbursement is usually long, lasting 1 or more years, in Eastern European countries [31]. This systemic limitation significantly impacts the outcomes of patients with refractory mCRC and should be explicitly acknowledged in regional treatment strategies. 

Among the currently sparse real-world data assessing the safety and effectiveness of fruquintinib, data from the Eastern European regions remain scarce. This highlights the urgent need to generate region-specific real-world evidence through robust cancer registries and observational studies to better inform clinical practice and policy decisions. Furthermore, with the emergence of therapies featuring novel mechanisms of action and MDT approaches in oncology care, there is an urgent need for medical education for all healthcare professionals involved in oncology care across this region. The experts from Bosnia and Herzegovina, Montenegro, and Hungary emphasised the need for learning activities to educate oncologists and healthcare professionals about the role of fruquintinib in later-line mCRC treatment.

Many experts also highlighted the limited opportunities for clinical trial enrolment in the region as a limitation hindering patient access to innovative therapeutic agents and depriving healthcare professionals of critical experience with novel treatment modalities. The development of systematic early access frameworks, alongside strengthening participation in both global and regional clinical trials, would not only enhance patient outcomes but also expedite the accumulation of real-world evidence.

## Future prospects

Owing to its acceptable safety profile along with significant survival benefits, a prospective application of fruquintinib at an earlier stage could be considered within the therapeutic regimen for mCRC. Moreover, the experts expressed a considerable degree of interest regarding the potential of combining fruquintinib with established chemotherapy regimens and targeted therapeutic agents, particularly in patients carrying RAS mutations and those exhibiting liver metastasis, highlighting the need for tailored approaches in this specific patient population. In light of these observations, conducting clinical trials alongside the collection of real-world data is imperative, as it is essential to substantiate the utilisation of such agents and to thoroughly investigate the optimal sequencing of treatments, thereby emphasising the critical necessity for additional research in this domain. The experts underscored the substantial importance of ongoing education and training for oncologists concerning the rapidly evolving treatment landscape, which now increasingly incorporates the role of novel therapeutic agents such as fruquintinib. Furthermore, the establishment of national registries coupled with structured systems for the collection of real-world data has been identified as a pivotal enabler that can facilitate evidence-based decision-making and bolster advocacy efforts for policy changes in the realm of oncology. The acceptance of new pharmacological agents such as fruquintinib by the National Health Insurance Funds and the development of effective financing strategies are of paramount importance for ensuring the successful adoption and integration of these innovative drugs into standard treatment protocols. The lack of early access programmes highlights a significant shortfall in providing patients with timely access to innovative therapeutic options. Expanding these programmes in the future could significantly enhance treatment opportunities for eligible individuals. Lastly, linking reimbursement to data reporting could further incentivise comprehensive outcomes, thus enhancing the overall quality of cancer care.

The experts expressed optimism about exploring fruquintinib in earlier treatment lines and in combination with chemotherapy or targeted agents, particularly in patients with RAS-mutated or liver-dominant mCRC. However, these strategies remain investigational, and there is a paucity of region-specific clinical trials to validate these approaches. The lack of large-scale real-world evidence from Eastern Europe limits the ability to define optimal sequencing and patient selection. Expanding clinical trial participation and generating local data were identified as critical steps in bridging this evidence gap.

## Conclusion

The virtual meeting offered valuable insights into the treatment patterns for mCRC and the associated challenges encountered in Eastern Europe based on the views of experts from Bosnia and Herzegovina, Bulgaria, Croatia, Hungary, Latvia, Lithuania, Montenegro, Romania, Serbia, and Slovenia. A significant disparity in the economic status exists between Eastern European countries and their Western European counterparts, leading to disparities in access to healthcare and reimbursement policies. The experts identified several challenges in managing patients with mCRC in this region, including the need for molecular and genetic profiling, multidisciplinary coordination, limited availability of and access to targeted treatments, and financial constraints due to reimbursement issues. Delays in the access to and availability of newly approved medications, as well as delays in reimbursements, may postpone treatment initiation and 0complicate timely decision-making for targeted therapies. The experts generally emphasised the need for expedited access to molecular genetic tests, reimbursement for various cancer treatments, expanded access programmes for new medications, and the importance of multidisciplinary approaches in managing mCRC as key strategies for improving therapeutic outcomes. Additionally, the experts highlighted the importance of patient education and participation in both global and regional clinical trials. They also noted the need for generating real-world evidence on the safety and efficacy of fruquintinib, its comparative efficacy versus other later-line therapies, its efficacy as an earlier line of therapy, and its sequencing in the current treatment practices. Furthermore, despite the lack of direct experience with fruquintinib, most experts acknowledged its potential efficacy as a later-line therapy for mCRC and considered its effectiveness to be comparable to that of other third-line treatments.

## Data Availability

All transcripts of the questions, insights, and comments are available at Takeda d.o.o. and from the corresponding author.

## Abbreviations

1L: First line of therapy
2L: Second line of therapy
3L: Third line of therapy
4L: Fourth line of therapy
AE: Adverse event
BRAF: V-Raf murine sarcoma viral oncogene homologue B
CA19-9: Carbohydrate antigen 19–9
CEA: Carcinoembryonic antigen
CRC: Colorectal cancer
CT: Computed tomography
dMMR: Deficient mismatch repair
DPYD: Dihydropyrimidine dehydrogenase
EAP: Early access programme
ECOG: Eastern Co-operative Oncology Group
EGFR: Epidermal growth factor receptor
ESMO: European Society for Medical Oncology
FOLFIRI: Folinic acid, fluorouracil, and irinotecan
FOLFOX: Folinic acid, fluorouracil, and oxaliplatin
FGFR: Fibroblast growth factor receptor
HER2: Human epidermal growth factor receptor 2
IHC: Immunohistochemistry
KIT: Proto-oncogene c-kit
KRAS: Kirsten rat sarcoma viral oncogene homologue
mCRC: Metastatic colorectal cancer
MDT: Multidisciplinary team
MMR: Mismatch repair
MSI: Microsatellite instability
NGS: Next-generation sequencing
NHIF: National Health Insurance Fund
NRAS: Neuroblastoma rat sarcoma viral oncogene homologue
OS: Overall survival; PCR, polymerase chain reaction
PFS: Progression-free survival
RAS: Rat sarcoma
RET: Rearranged during transfection
TAT: Turnaround time
UGT1A: Uridine diphosphate glucuronosyltransferase family 1 member A
VEGFR: Vascular endothelial growth factor receptor
WD: Work day
